# Development of Dual-Function Microelectronic Fibers for pH and Temperature Sensing: Toward *In Vivo* and Wearable Applications

**DOI:** 10.1021/acsmeasuresciau.4c00092

**Published:** 2025-03-06

**Authors:** Mahiro Kubo, Mayuko Abe, Etienne Le Bourdonnec, Sheau-Chyi Wu, To-En Hsu, Takao Inoue, Yuanyuan Guo

**Affiliations:** † Department of Materials Science and Engineering, School of Engineering, Tohoku University, Sendai, Miyagi 980-8579, Japan; ‡ Department of Earth Science, School of Science, Tohoku University, Sendai, Miyagi 980-8579, Japan; § Frontier Research Institute for Interdisciplinary Sciences (FRIS), Tohoku University, Sendai, Miyagi 980-0845, Japan; ∥ College of Engineering, Chang Gung University, Guishan District, Taoyuan 33302, Taiwan; ⊥ Organization of Research Initiatives, Yamaguchi University, Yoshida, Yamaguchi 753-0841, Japan; # Graduate School of Biomedical Engineering, Tohoku University, Sendai, Miyagi 980-8579, Japan

**Keywords:** thermally drawn fibers, dual-function, microthermocouple, pH sensing, polyaniline electropolymerization

## Abstract

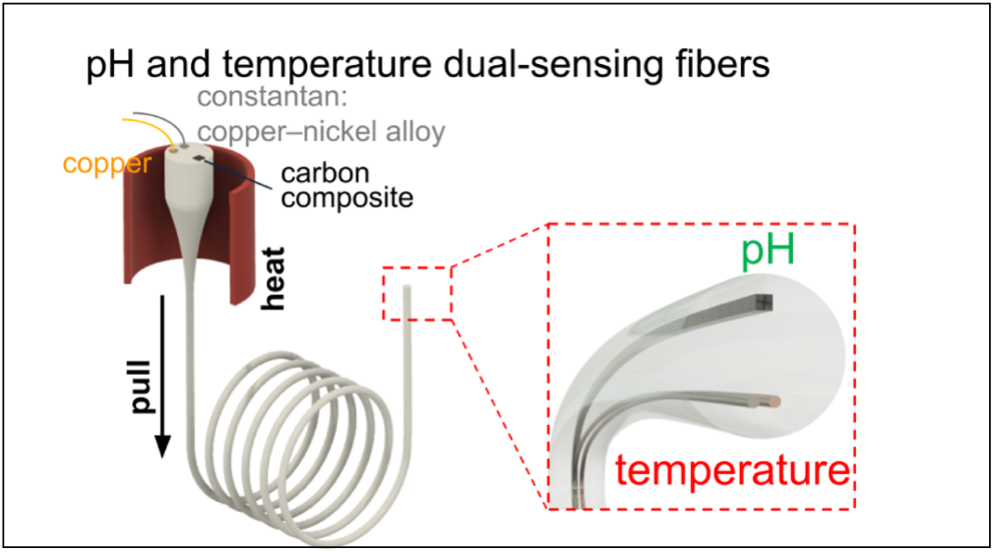

Temperature plays a crucial role in biological functions in normal physiological and pathological states and is intricately linked with chemical dynamics *in vivo* at the cellular, circuit, and system levels. Despite advances in temperature measurement technologies for internal monitoring, systems capable of simultaneously tracking localized temperature and chemical changes are still underdeveloped. In this study, we introduce dual-sensing hybrid fibers with a miniature footprint of <400 μm in diameter, fabricated using the thermal drawing process. These fibers exhibit precise temperature sensitivity, detecting changes as small as 0.5 °C, and demonstrate highly sensitive and reversible pH detection, a critical physiological parameter. Furthermore, through laser micromachining and surface functionalization, we highlight the potential of these fibers for wearable applications in dual pH and temperature sensing. This innovative dual-sensing technology offers a versatile platform for probing temperature and chemical signaling *in vivo* and wearable applications, with significant implications for therapeutic development and a deeper understanding of biological processes in various environments.

## Introduction

Temperature and chemical signaling are integral to physiological processes in living organisms, playing a crucial role in maintaining homeostasis, regulating bodily functions, and responding to environmental changes.^1^ These dynamics are fundamental in the brain and influence cognitive functions, emotions, and behaviors.^2^ However, studying these signals, especially within the brain, has been challenging due to the limitations of existing monitoring technologies.^3^ Traditional noninvasive methods—such as microwave radiometry,^4^ magnetic resonance thermometry,^5^ ultrasound thermometry,^6^ and near-infrared spectroscopy^7^—can provide accurate temperature measurements but suffer from poor spatial resolution, cumbersome setups, and an inability to simultaneously track multiple modalities.

In response to these limitations, recent advancements have led to the development of microscale implantable devices capable of locally monitoring temperature,^8,9^ while also integrating other functionalities such as electrical stimulation, electrophysiological recordings, and optogenetics.^10^ These devices are typically based on planar structures on silicon or flexible substrates using established photolithography and printing technologies. However, they often rely on thermistors for thermal monitoring, which, despite being easy to integrate, have several drawbacks, including slow response times, limited durability, and complex calibration requirements.^8,11^ Moreover, although silicon-based neural probes have advanced significantly, they have not yet successfully integrated chemical sensing with temperature monitoring, highlighting the critical need for dual-sensing probes that can track both thermal and chemical signals *in vivo*.

Recent progress in polymer fiber-based multifunctional neural probes, fabricated through the thermal drawing process, has opened new avenues for multimodal sensing.^10,12^ This process, long used in the telecommunications industry to produce optical fibers, allows for the integration of various functionalities into a single preform, which is then drawn into microscale fibers that retain the preform’s characteristics. These fibers have been applied in neuroscience to explore neural dynamics across electrical, optical, and chemical modalities.^13,14^ However, monitoring thermal dynamics in conjunction with chemical sensing remains unexplored.

**Figure 1:**
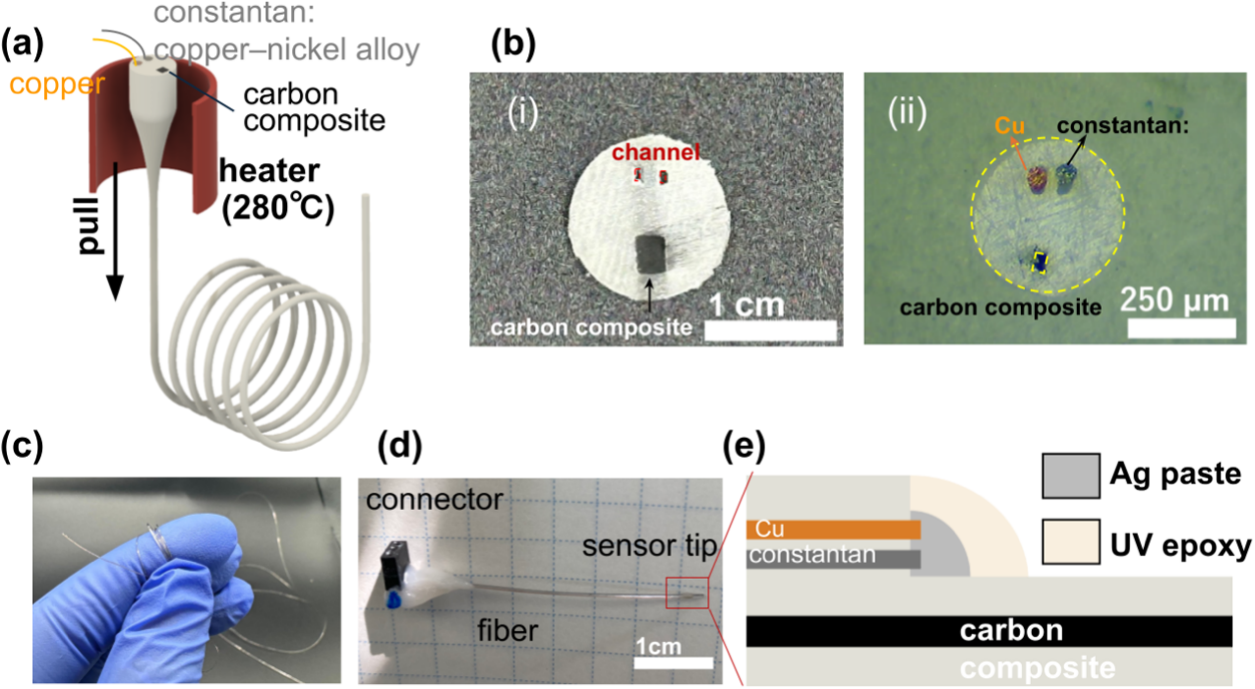
Thermally drawn temperature and chemical fiber sensor. (a) Schematic of the thermal drawing process used to fabricate the fiber. (b) (i) Cross-sectional photo of the preform and (ii) microscopic image of the fiber cross-section after drawing. (c) Demonstration of the fiber’s flexibility. (d) Image of the dual-sensing probe-type device constructed from the fiber. (e) Schematic illustration of the dual-sensing device tip, highlighting the integration of temperature and chemical sensing elements.

In this study, we developed a dual-sensing fiber, with a miniature footprint of <400 μm in diameter (Figure 1a,b), that utilizes thermocouple principles based on the Seebeck effect for precise temperature sensing^15^ and electrochemical methods for pH detection.^16−19^ By leveraging the thermal drawing process, we integrated two thin metal wires, copper (Cu) and constantan (CuNi), into the fibers, enabling accurate and rapid temperature measurements (Figure 1a). In addition, we synthesized a 20% carbon nanofiber(CNF) composite and incorporated it into the same fibers,^12,20^ creating an enhanced electrochemical sensor (Figure 1b). Electrode functionalization was achieved through the electropolymerization of polyaniline (PANI),^16,19^ a conductive polymer that binds specifically to protons, enabling efficient pH sensing. We performed extensive characterization of both the thermal and chemical sensing capabilities in an *in vitro* setting, demonstrating their high precision and potential for future *in vivo* applications.

Beyond *in vivo* use, we further enhanced the multiwire for dual sensing with the potential in wearable health surveillance applications by incorporating laser micromachining and surface functionalization techniques. These modifications enable longitudinal dual-sensing of pH and temperature along the fiber, which can be further developed in a wearable textile format, offering new possibilities for continuous physiological monitoring in real-world settings.

## Materials and Methods

### Chemicals

Aniline, phosphate-buffered saline (Dulbecco’s PBS), and 1 M sulfuric acid were procured from FUJIFILM Wako Pure Chemical Corporation, Japan. Agarose and pH buffer solutions were obtained from Sigma-Aldrich Japan Corporation and Monotaro, respectively. Ag paste was purchased from SPI Supplies. UV epoxy was purchased from SeaForce Inc. Carbon grease (CG, particle size of 40 nm) was purchased from KITACO Co., Ltd. (Japan).

### Instruments

The Gamry Interface 1010E and PalmSens4 were utilized as potentiostats for pH measurements. A laser marking system (LaSoX, SeaForce, Japan) was used to expose the wires within the PC cladding. For all cross-sectional observations and analyses, the Stemi 305 stereomicroscope and Labscope software, both from ZEISS, were employed. For the microstructural analysis, scanning electron microscopy (SEM, HITACHI) was used. Fiber fabrication was done with a customized thermal drawing system. Thermal sensing was performed using a CTU-Mini thermal bath (TAITEC Online) and pH of buffer solutions was verified via a pH meter (LAQUAtwin, AS-ONE). The surface of carbon composites of dual-sensing hybrid fiber was pretreated with an air plasma from STREX, Inc.

### Fabrication of Dual-Sensing Hybrid Fiber

A 20 wt % carbon nanofiber (CNF) composite was synthesized following methods established in previous studies.^12,20^ The composite was formed into a slab measuring 2.7 × 2.7 × 100 mm^3^. A slot, matching the cross-sectional dimensions of the CNF slab (3 × 3 mm^2^), was machined into a polycarbonate (PC) rod (provided by Hakudo Corp.) with a diameter of 8 mm. The CNF slab was then inserted into this slot. Subsequently, two additional slots, each measuring 1 × 1.2 × 100 mm^3^, were machined to accommodate the metal wires. The rod was then wrapped with PC films of 100 μm thickness until it reached a final diameter of 14 mm. This assembly was consolidated at 195 °C under a vacuum to form a preform. During the drawing process, copper and constantan wires (supplied by Nilaco Corp.) were simultaneously fed passively into the two machined channels (Figure 1a). The preform (Figure 1b(i)) was then heated to 280 °C and drawn into fibers (Figure 1b(ii)) using a custom-built table-top fiber drawing system. The bait-off was set to 75 g, and the drawn fiber was in decades of centimeters (Figure 1c).

### Fabrication of Probe-Type Dual-Sensing Device

The fiber tip was freshly cut using a razor blade. For the thermal sensing junction at the tip, both the copper (Cu) and constantan wires were exposed by using a surgical blade, approximately 5 mm from the tip. These exposed ends were then joined using silver paint to establish an electrical contact (Figure 1e). This joint was subsequently protected with epoxy to ensure its durability and stability. To make electrical connections with external equipment, the ends of the Cu and CuNi wires, along with the CNF-composite electrode at the opposite ends of the fiber, were exposed. These exposed sections were connected to pin connectors by using silver paint to secure electrical continuity. The connections were then further encapsulated in epoxy to safeguard against mechanical stress and environmental factors (Figure 1d).

### Evaluation of Thermal Sensing with the Dual-Sensing Hybrid Fiber

**Figure 2:**
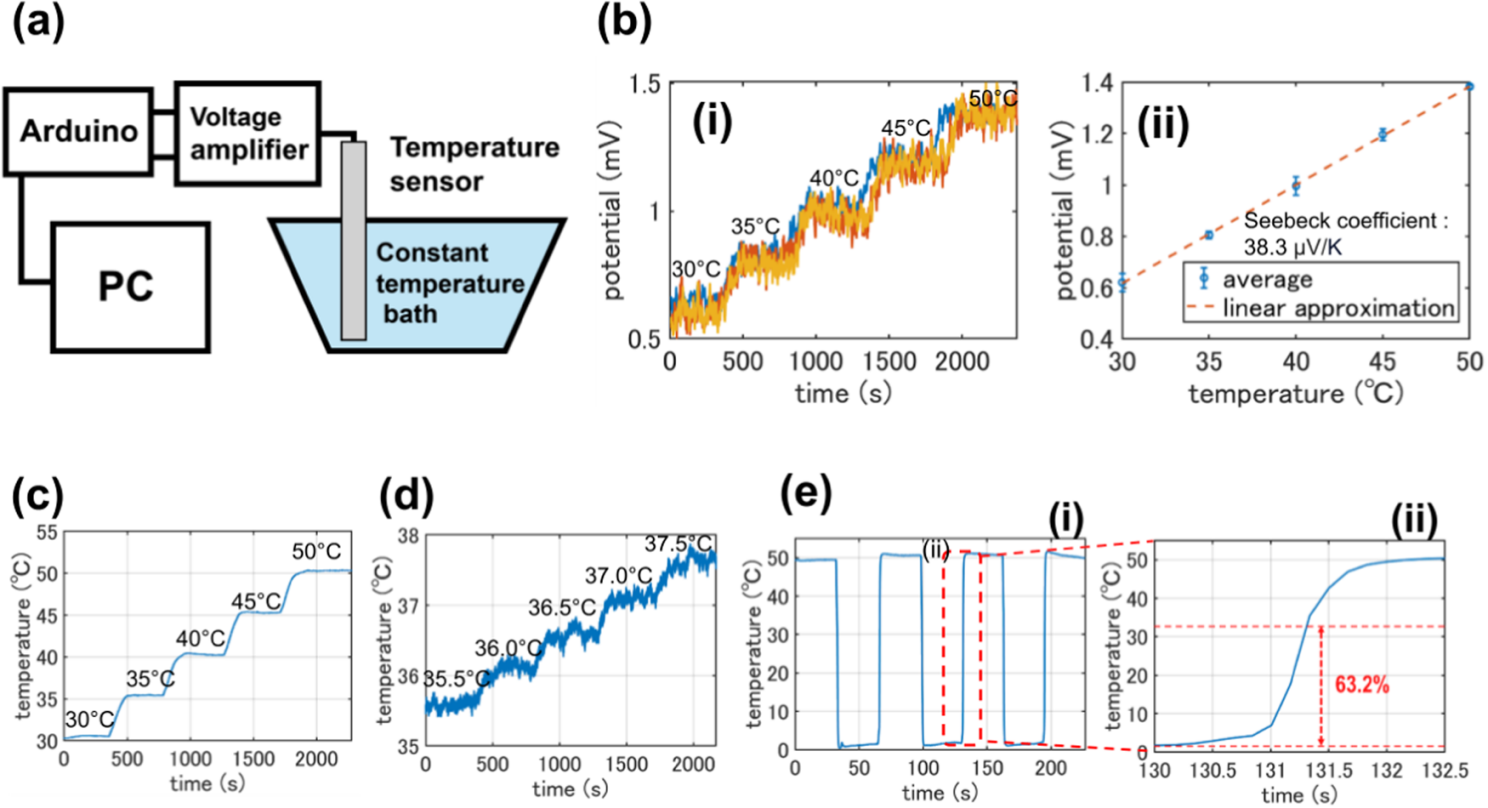
Characterization of the thermal sensing capabilities of the dual-sensing fiber. (a) Schematic of the experimental setup used for thermal characterization. (b) (i) Continuous recording of the potential difference generated between two wires in response to temperature changes, and (ii) correlation between the potential difference and temperature, verifying the Seebeck effect. (c) Continuous recording of the fiber’s thermal response with temperature increments of 5 °C. (d) Continuous recording of the fiber’s thermal response with finer temperature increments of 0.5 °C. (e) (i) Time response of the fiber during thermal sensing and (ii) time scale zoomed-in view of the dashed area in (e(i)), demonstrating the speed of temperature detection.

Thermal sensing was conducted using a programmable temperature water bath (CTU-Mini, TAITEC Online) (Figure 2a). The hybrid device was enclosed within a vial filled with PBS solutions and subjected to a controlled temperature increase of 30–50 °C in increments of 5 °C and later with finer temperature increments of 0.5 °C. Temperature measurements were collected directly through the fiber, using a specific setup involving a MAX31856 thermocouple to digital converter and an Arduino. In addition, a custom designed circuit was implemented to convert the temperature readings into electrical potential (Figure S1), facilitating the verification of the Seebeck effect principle utilized in our dual-sensing hybrid fibers.

### Electropolymerization of PANI on the Carbon Electrode of the Hybrid Fiber

**Figure 3:**
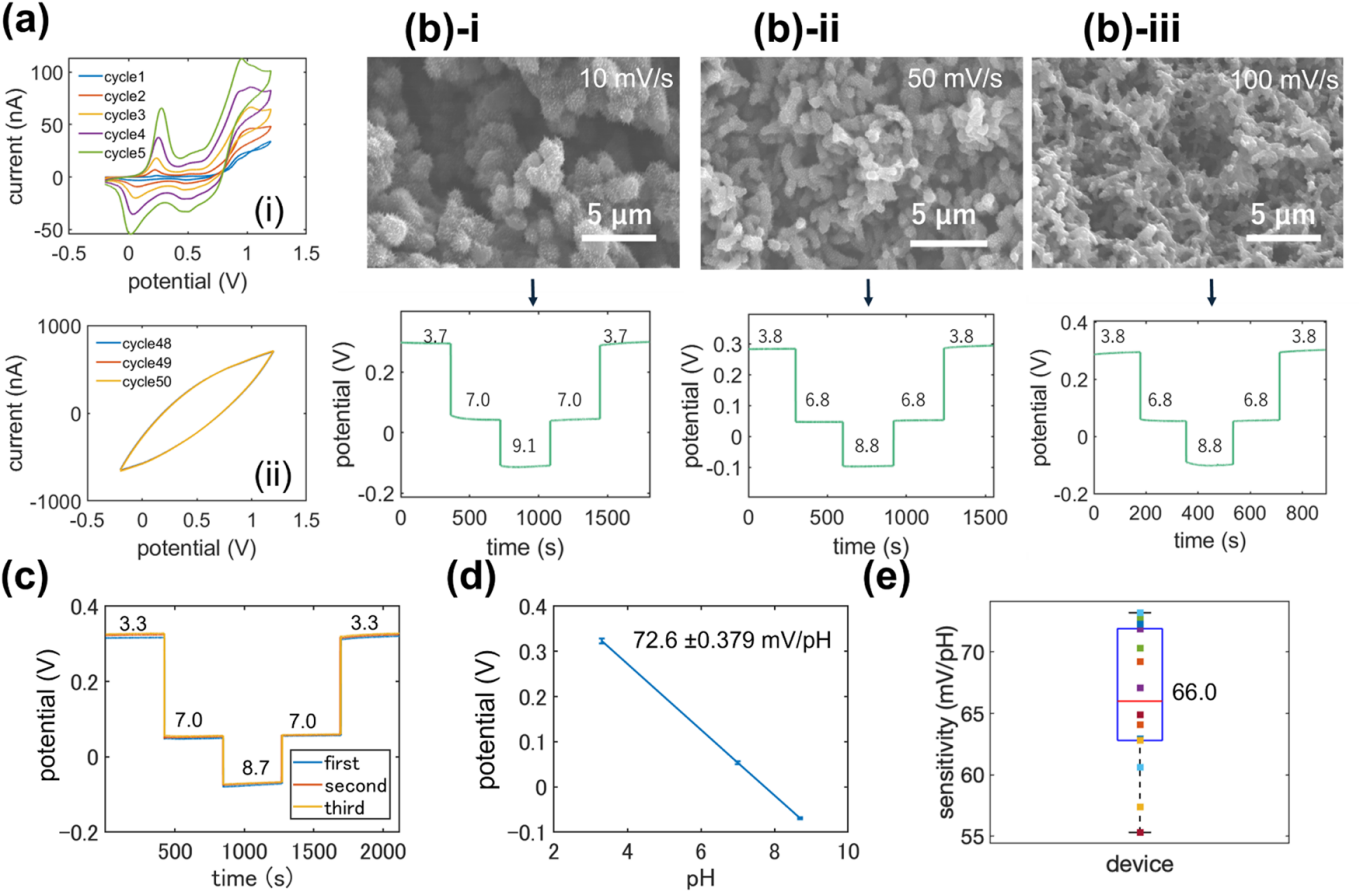
Characterization of the pH sensing capabilities of the dual-sensing fiber. (a) Electropolymerization of PANI on the fiber: (i) initial cycles and (ii) final cycles of the process. (b) Influence of the PANI microstructure on pH-sensing performance: comparison of cyclic voltammetry at different scanning speeds (10, 50, and 100 mV/s), the pH values of buffer solutions are indicated in the pH-sensing characterization. (c) Repeated continuous pH-sensing measurements of the PANI-coated fiber with pH values of buffer solutions indicated in the figure. (d) The corresponding average response of (c). (e) Cross-device consistency: devices coated under the same conditions (50 mV/s) exhibited similar super-Nerstian sensitivity with the median at 66.0 mV/pH.

Initially, the fiber tip or the carbon filling of fiber was subjected to plasma treatment for 20 s to activate the surface. Aniline (0.1 M) was dissolved in sulfuric acid (1 M) and stirred by using a vortex mixer until a clear solution was obtained. Electropolymerization of PANI on the carbon electrode was performed using a three-electrode system via a potentiostat, which included a platinum wire as the counter electrode and a Ag/AgCl electrode (3 M NaCl) as the reference electrode. Cyclic voltammetry was employed to facilitate polymerization, with the potential ramping from −0.2 to 1.2 V (Figure 3a). This process was carried out at scan speeds of 10, 50, and 100 mV/s over 50 cycles (Figure 3b). All procedures were carried out under ambient conditions.

### Evaluation of the pH Sensing with the Dual-Sensing Hybrid Fiber

The pH-sensing capabilities of the hybrid fiber were evaluated by using a two-electrode configuration with a potentiostat (PalmSens4 or Gamry Interface 1010E). A saturated Ag/AgCl reference electrode was employed, maintained at a stable potential through a salt bridge that connects the reference electrode in the PBS solution to various pH test solutions.^21^ The open circuit potential between the hybrid fiber and the reference electrode was systematically recorded in a range from acidic to alkaline solutions(Figure 3b). To evaluate the reversibility of pH-sensing, the pH of the solutions was gradually changed from acid to alkali then back to acid. This procedure allowed for the demonstration of the response consistency and reversibility of pH sensing over multiple cycles. In addition, the pH value of the buffer solution was verified via a pH meter to ensure accuracy. In addition, to evaluate its selectivity, a single PANI-functionalized fiber was subject to the selectivity measurement of Na^+^ or K^+^ for 4 times. In these experiments, NaCl and KCl solutions were prepared over concentrations of 0.1 1, 10, and 100 mM (Figure S3). We also characterized the ion selectivity of PANI-coated hybrid fibers in 10 mL of PBS solutions with the addition of 1 mL of 10 mM NaCl, 10 mM KCl and pH 4 buffer solutions (Figure S4). Furthermore, we recorded the voltage drift of our fiber sensor at pH buffers with constant pH over extended time of 2 h (Figure S5) and evaluated their long-term pH sensitivity changes over days for multiple devices (Figure S6).

### Simultaneous pH and Temperature Sensing Characterization with the Dual-Sensing Device

**Figure 4:**
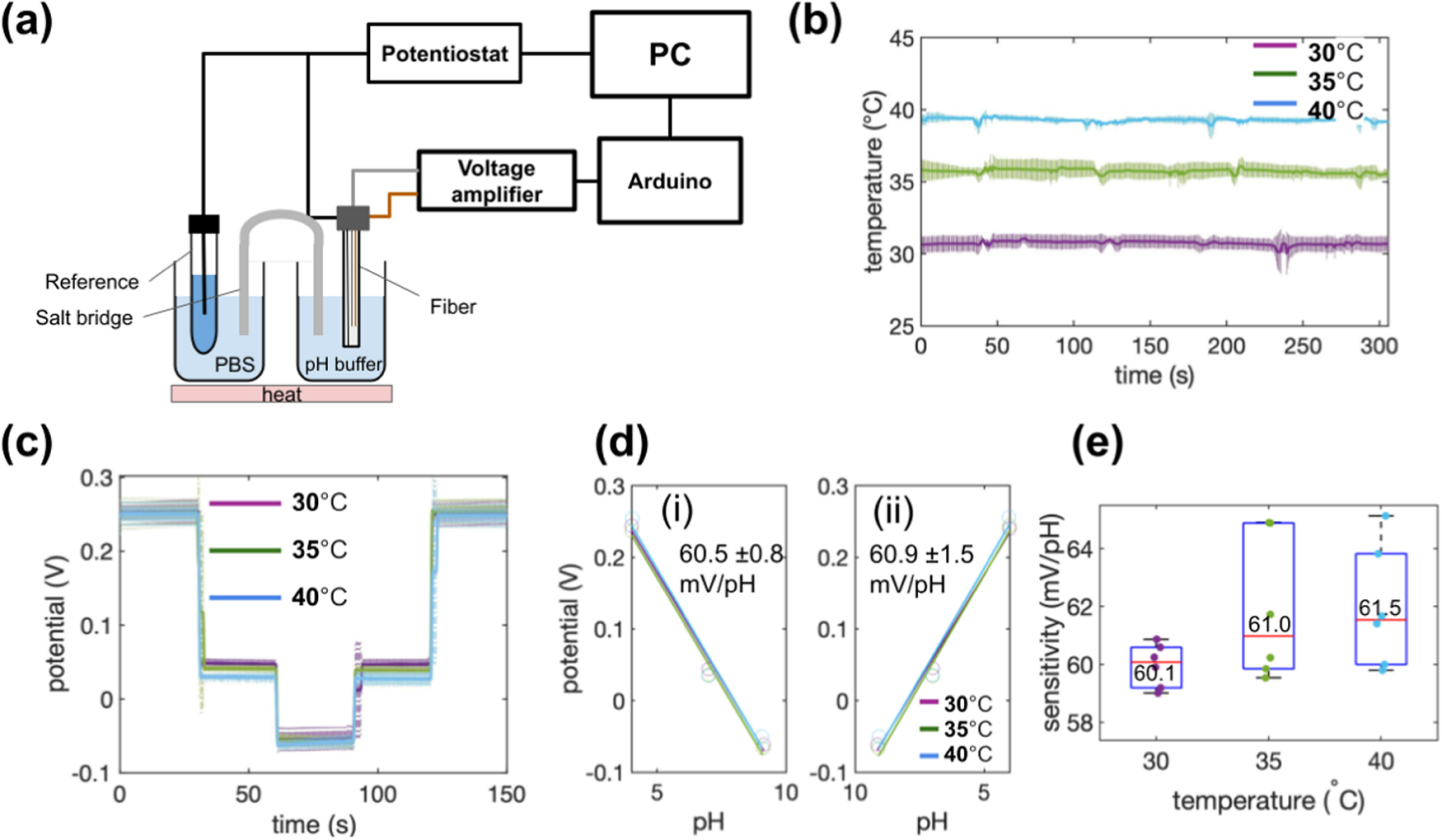
Simultaneous characterization of the temperature and pH-sensing capabilities of the dual-sensing fiber. (a) Schematic of the experimental setup for simultaneous temperature and pH measurements. (b) Continuous temperature monitoring, with the shaded area representing the standard deviation. (c) Simultaneous pH measurements at different temperatures, with the shaded area indicating the standard deviation. (d) Calibration curves for pH sensing at various temperatures: (i) during increasing pH levels and (ii) during decreasing pH levels. (e) Correlation between pH sensitivity and temperature changes.

Simultaneous sensing experiments were conducted using a programmable temperature water bath to host both pH buffer solutions and PBS solutions (Figure 4a). pH measurements were systematically recorded across a range of values, starting from pH 4, increasing to 7 and 9, then reversing back to 7 and finally to 4. Concurrently, temperature readings were taken to assess the sensor’s performance under varying thermal conditions. Initially, the temperature of the solutions was stabilized at 30 °C, during which the pH-sensing experiments were carried out (Figure 4b,c). Subsequently, the temperature was incrementally raised to 35, 40, 45 °C, and finally 55 °C, with pH measurements recorded at each of these temperature points to evaluate the relationship of the pH sensing with the temperature control.

### Fabrication of Multiwire Fiber

**Figure 5:**
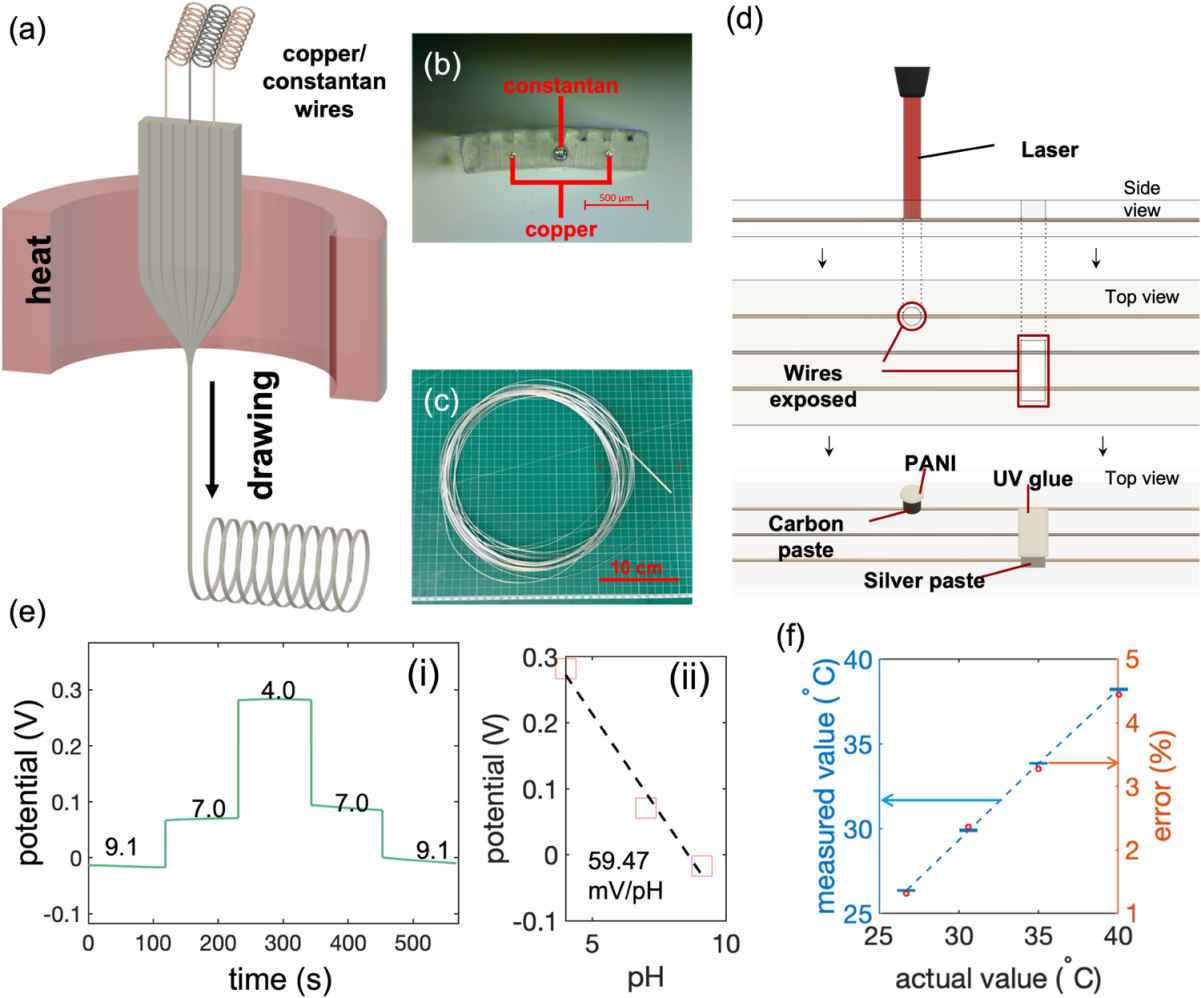
Dual-sensing fiber for textile-based wearable applications. (a) Schematic of the thermal drawing process incorporating multiple wires. (b) Cross-sectional view of the fiber showing three embedded wires and surface grooves for sweat collection. (c) Image of the thermally drawn, elongated fibers. (d) Laser micropatterning along the fiber’s longitudinal surface followed by surface functionalization. (e) Continuous pH sensing: (i) real-time pH measurements of pH buffer solutions and (ii) corresponding sensitivity analysis. (f) Characterization of the side-mounted thermocouple integrated within the fibers for temperature sensing.

One PC plate measuring 200 mm × 24 mm × 6 mm was machined with three grooves to accommodate metal wires. An additional PC plate of the same dimensions was machined with multiple surface grooves. The two PC plates were then consolidated at 180 °C by using a hot press. Following consolidation, the assembly was drawn into fibers using a customized thermal drawing tower, during which two copper wires and one constantan wire were passively embedded into the fibers drawn into decades of meters (Figure 5a–c).

### Fabrication and Characterization of Wearable-type Dual-Sensing Device

The wires within the multiwire fiber were exposed using laser micromachining.^22^ Carbon grease was applied to fill one of the exposed copper wire holes on which PANI was electropolymerized as pH sensors using the aforementioned method. Silver paste was used to connect the copper and constantan wires to form a thermocouple junction, and the junction was subsequently sealed with UV epoxy (Figure 5d). The opposite ends of the wires were exposed and connected to pins through soldering, ensuring electrical connectivity. The pH sensing and thermal sensing performance was characterized using the same method as the probe-type device.

## Results and Discussion

### Thermal Sensing Characteristics of the Fiber-Based Dual-Sensing Hybrid Device

The thermocouple at the tip of the hybrid device was calibrated in a temperature-controlled water bath with temperatures ranging from 30 to 50 °C with a step of 5 °C and 35.5 to 37.5 °C with a step of 0.5 °C respectively. Initially, the Seebeck effect was verified by using a custom-built voltage amplifier circuit (Figure S1), where the potential developed at the wire terminals was recorded in relation to temperature changes over time (Figure 2b(i)). Repeated measurements demonstrated the stability of the device’s thermal sensing. Furthermore, the potential difference between the Cu and CuNi wires was calculated during these temperature changes. Through linear regression fitting, the slope was determined as 38.3 μV/K to closely match the theoretical value (39.9 μV/K) of the Seebeck constant for these materials (Figure 2b(ii)). Following verification of the Seebeck effect, a commercial amplifier system (thermocouple amplifier MAX31856) was utilized to minimize noise. The thermal sensing performance is shown in Figure 2c. Given the importance of high-precision temperature measurement for biological applications, the resolution of temperature detection was tested down to 0.5 °C, confirming its capability for precise detection. Not only is high resolution important but the response time is also critical for *in vivo* temperature sensing. Temperature-sensing cycles were conducted in 50 °C water and ice water (Figure 2e). Considering the negligible time delay during device transfer between water baths, the device’s response time was calculated based on the time required to reach 63.2% of an instantaneous temperature change. This calculation, indicated as 37.0 ± 5.5 and 28.0 ± 4.0 ms for rising and falling times, provides detailed time resolution for high-precision temperature measurements.

### pH Sensing via the PANI Electropolymerization on Fiber-Based Dual-Sensing Hybrid Devices

We further characterized the pH-sensing performance of the PANI-coated hybrid devices. Figure 3a,b presents the typical cyclic voltammogram (CV) for PANI electropolymerization on the carbon composite electrodes of hybrid devices over 50 cycles. Initially, clear reactions of PANI with three distinct peaks were observed. The first pair of redox peaks corresponds to the interconversion between the leucoemeraldine (LE) state and the half-oxidized emeraldine base (EB) state of PANI. The third pair of redox peaks results from the oxidation of EB to the fully oxidized pernigraniline (PN) state and vice versa. The middle second redox peaks, attributable to the oxidation of segments of PANI chains into benzoquinone (Bq) species, tend to disappear after several successive scans (Figure 3b).^23^ To investigate the impact of scanning rate on both the microstructures and sensing performance, we varied the rate from 10 to 50 mV/s and then to 100 mV/s. This adjustment allowed us to observe the evolution of the PANI microstructures from fine-particle-like structures to rod-like structures and finally to coarsened, line-connected membranes. Then we used the two-electrode configuration to characterize the pH-sensing performances, to minimize the influence of junction potential developed at the reference electrode with the pH buffer solution, we introduced the salt bridge into the measurement system (Figure 3a).^21^ In addition, during the measurement, we used a pH meter (As-ONE LAQUAtwin, AS-pH-22) to verify the pH of the buffer solutions. Though all structural variations show similar pH-sensing behaviors, the stability of the mixtures may vary. The balance between the fine microstructure and coating yield has to be made. So to ensure adequate time for aniline diffusion and proper polymerization on the carbon electrode surface, we standardized the scanning rate at 50 mV/s. Subsequent stability tests and statistical analysis demonstrated a robust performance. Figure 3c details the pH-sensing performance with PANI coated at a scanning speed of 50 mV/s, showing high stability and sensitivity in repeated measurements (72.6 ± 0.379 mV/pH) (Figure 3d), exerting super-Nerstian behavior consistent with previous studies.^16,24^ Moreover, cross-device consistency (*N* = 14) was evaluated, as shown in Figure 3e, where devices coated under the same conditions (50 mV/s) exhibited similar super-Nerstian sensitivity with the median at 66.0 mV/pH and the average 66.05 ± 5.84 mV/pH, a close value in median and average showing that pH sensitivity variations of cross devices are rather symmetrical.

For Nerstian equations,
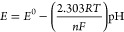
1where *E* is the measured potential (V); *E*^0^ is the standard electrode potential (V); *R* is the universal gas constant (8.314 J·mol^–1^·K^–1^); *T* is the absolute temperature (K); *n* is the number of electrons transferred (for pH sensing, *n* = 1); *F* is the Faraday constant (96,485 C·mol^–1^); and pH is the hydrogen ion concentration, expressed as −log[H^+^].

The Nernstian slope, i.e., the pH sensitivity under standard conditions (25 °C or 298 K) is
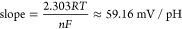
2

Madeira et al. examined the impact of surface hydration on super-Nernstian sensitivity in PANI-based pH sensors,^24^ explaining surface hydration enhances the mobility of ions, enabling faster protonation and deprotonation reactions. To account for surface hydration, they incorporated a factor 1/α, which represents the hydrogen ion/transferred electron ratio as the result of the enhancement of the effective protonation due to the hydrated surface. The modified Nernst equation is
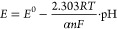
3

The observed slope of the pH response (under standard conditions (25 °C or 298 K)) is thus given by
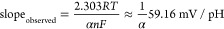
4

When 1/α is greater than 1, it indicates that the surface hydration is enhancing the protonation and deprotonation kinetics, resulting in a more sensitive pH response, which is beyond what is predicated by the standard Nerstian equation.

Additionally, the selectivity of the PANI-functionalized fiber electrode was evaluated, showing no linear response to Na^+^ and K^+^ ions (Figure S2). Furthermore, the long-term stability assessment demonstrated that the PANI-coated fiber electrode maintained high stability over several weeks (Figure S3).

### Simultaneous Detection of pH and Temperature via the Dual-Sensing Device

We demonstrated the capability of our fiber-based dual-sensing device for the simultaneous detection of pH and temperature. The open circuit potential corresponding to the pH response was recorded by using a potentiostat, while temperature measurements were obtained via customized electronics, as depicted in Figure 4a. The results in Figure 4b confirm reliable temperature sensing behavior. Additionally, continuous recording of open circuit potentials in response to freshly prepared solutions of varying pH levels (pH 4,7, and 9) exhibited negligible deviation when performed at physiological temperatures of 30, 35, and 40 °C (Figure 4c). Figure 4d shows that pH sensing exhibited excellent reversibility, indicating that the PANI coating remains stable across the physiological temperature range. A detailed analysis of the temperature-dependent pH sensitivity further revealed an increase in pH sensitivity at higher temperatures (Figure 4e), consistent with Nernstian behavior, as explained in eqs 2 and 4.

### Potentials for Wearable Applications

In addition to the application of the miniature probe, we demonstrated the potential of our dual-sensing fibers for wearable pH and temperature sensing, as shown in Figure 5. A multiwire fiber with surface grooves for potential sweat collection was fabricated using thermal drawing (Figure 5a), successfully integrating Cu and CuNi within thin fibers, which can be developed as sensors (Figure 5b). The metal wires were used because they can conduct signals along a prolonged fiber (Figure 5c) length with low loss. Laser micromachining was employed to selectively expose the wires along the longitudinal surface of the fiber at designated positions (Figures 5d and S4). One Cu wire was functionalized with carbon paste, which was subsequently electropolymerized with PANI. Additionally, one Cu wire was fused with CuNi with silver paste, followed by UV glue encapsulation, to create a thermocouple. We demonstrated excellent pH-sensing behavior with high sensitivity and reversibility, as shown in Figure 5e. For temperature sensing, accurate measurements were achieved within the skin temperature range of 25–40 °C (Figure 5f). These results highlight the potential of our dual-sensing fiber for wearable applications in the body.

## Conclusions

In this study, we successfully developed and characterized a probe-type dual-sensing fiber device with a miniature footprint of <400 μm in diameter, capable of simultaneously measuring pH and temperature with high sensitivity and stability, showing great potential for *in vivo* localized monitoring of physicochemical parameters. The integrated thermocouple based on the Seebeck effect at the fiber tip demonstrated reliable temperature sensing across a broad physiological range, with high precision down to 0.5 °C and rapid response times in the millisecond range, making it highly suitable for real-time biological applications. The PANI-coated carbon composite electrode exhibited robust pH-sensing performance via open circuit potential measurement with excellent reversibility, sensitivity, selectivity, and long-term stability. This dual-sensing fiber fulfills the requirements, in terms of sensitivity and time response, for minimally invasive *in vivo* measurements, and ongoing efforts are focused on its biological deployment to investigate temperature and chemical dynamics in the brain under pathophysiological conditions. Additionally, we are optimizing its flexibility to accommodate long-term implantations in other organs, where a softer approach is essential.

Our fabrication process, which integrates thermal drawing and laser micromachining, enabled longitudinal functionalities of the fibers, enhancing their applicability for wearable systems. The subsequent functionalization with carbon paste and PANI facilitated accurate and precise pH sensing. Furthermore, microthermocouples were developed on the fiber surface by fusing Cu and CuNi, demonstrating the fiber’s versatility for textile-based, dual-sensing systems. To demonstrate its potential for wearable applications, the current temperature error at higher temperatures, which can reach up to 5%, needs to be addressed. Further optimization of the fiber’s geometric design is essential to enhance its temperature-sensing accuracy and response.

In this study, we characterized the detailed performance of our dual-sensing fibers; we also plan to further apply this technology to real biological experiments to probe *in vivo* pH and temperature dynamics in animal models or wearable format on the human body. Looking ahead, our goal is to incorporate additional sensing and modulation modalities, such as multi-ion dynamics,^18^ electrophysiology, and optical or electrical stimulation, within the same fibers. In addition, we are continuing to explore the capabilities of the PANI-functionalized microelectrode to modulate localized pH levels in biological systems.^25^

In conclusion, this dual-sensing fiber represents a promising platform for *in vivo* research and for wearable devices. It has significant potential for applications in brain-body correlations and real-time health monitoring. Its compact and efficient design enables simultaneous multisignal detection, opening new avenues for biomedical research and therapeutic development.

## Data Availability

The orginial data are available upon request to the corresponding author.
