# Supplementary material for: Development of Dual-Function Microelectronic Fibers for pH and Temperature Sensing: Toward In Vivo and Wearable Applications
Source: ACS Meas Sci Au. 2025 Mar 6;5(2):208–15. doi: 10.1021/acsmeasuresciau.4c00092 (PMC12006951; doi:10.1021/acsmeasuresciau.4c00092)
Supplement: Supplementary file 1 — tg4c00092_si_001.pdf [file tg4c00092_si_001.pdf]

Development of Dual-Function Microelectronic Fibers for pH and Temperature Sensing: Toward In Vivo and Wearable Applications

Mahiro Kubo,[†] Mayuko Abe,[‡] Etienne Le Bourdonnec,[¶] Sheau-Chyi Wu,[§] To-En Hsu,[§] Takao Inoue,[‖] and Yuanyuan Guo[*,¶,⊥]

†*Department of Materials Science and Engineering, School of Engineering, Tohoku University, Sendai, Miyagi, 980-8579, Japan*

‡*Department of Earth Science, School of Science, Tohoku University, Sendai, Miyagi, 980-8579, Japan*

¶*Frontier Research Institute for Interdisciplinary Sciences (FRIS), Tohoku University, Sendai, Miyagi, 980-0845, Japan*

§*College of Engineering, Chang Gung University, Guishan District, Taoyuan City, 33302, Taiwan*

‖*Organization of Research Initiatives, Yamaguchi University, Yoshida, Yamaguchi, 753-0841, Japan*

⊥*Graduate School of Biomedical Engineering, Tohoku University, Sendai, Miyagi, 980-8579, Japan*

E-mail: yyuanguo@fris.tohoku.ac.jp

List of Figures

1 Schematic of the customized circuit for thermocouple voltage amplification and temperature compensation. 4

2 Our PANI-coated fibers for pH sensing in buffer solutions with pH values of 4, 7, and 9. The fibers exhibited a sensitivity of 68.6 mV/pH, along with a rapid response to changes in pH. 4

3 Characterization of ion selectivity of PANI-coated hybrid fibers. (a) Selectivity over Na^+. (b) Selectivity over K^+. The device in Figure 2 was subject to the selectivity measurement of Na^+ and K^+ for 4 times. In these experiments NaCl and KCl solutions were prepared over the concentrations of 0.1 mM, 1 mM, 10 mM, 100 mM. 5

4 Characterization of ion selectivity of PANI-coated hybrid fibers in phosphate buffer solutions (PBS, 10 mL) with the addition of 1mL of 10 mM NaCl, 10 mM KCl and pH4 buffer solutions (same device). The addition of NaCl and KCl had minimal influence on the recorded open-circuit potential, indicating that PANI does not bind to Na^+ or K^+ ions. The pH of the PBS solution, initially recorded as 7.3 using a pH meter, changed to 7.3, 7.5, and 6.3 upon the addition of NaCl, KCl, and pH 4 buffer solutions, respectively. Only the addition of pH4 buffer solutions evoked the OCP increase of 71.1 mV which corresponds to the measured pH changes as well as indicated by sensitivity(68.6mV/pH)) of the sensors. 6

5 Voltage drift behaviors of our PANI-coated fibers in different pH buffer solutions of pH4 (purple), pH7 (green) and pH9 (blue) over 2 hours. 7

6 Long-term stability of PANI-coated hybrid fibers across different devices (N=3). (a) Example devices with pH sensing measurements and sensitivity over several days. (b) Sensitivity of multiple devices across different days. 7

7 The schematics of the laser micromachining and surface functionalization of multi-wire fibers are shown in (a-b), along with microscopic images of the fiber after laser exposure and subsequent functionalization. 8

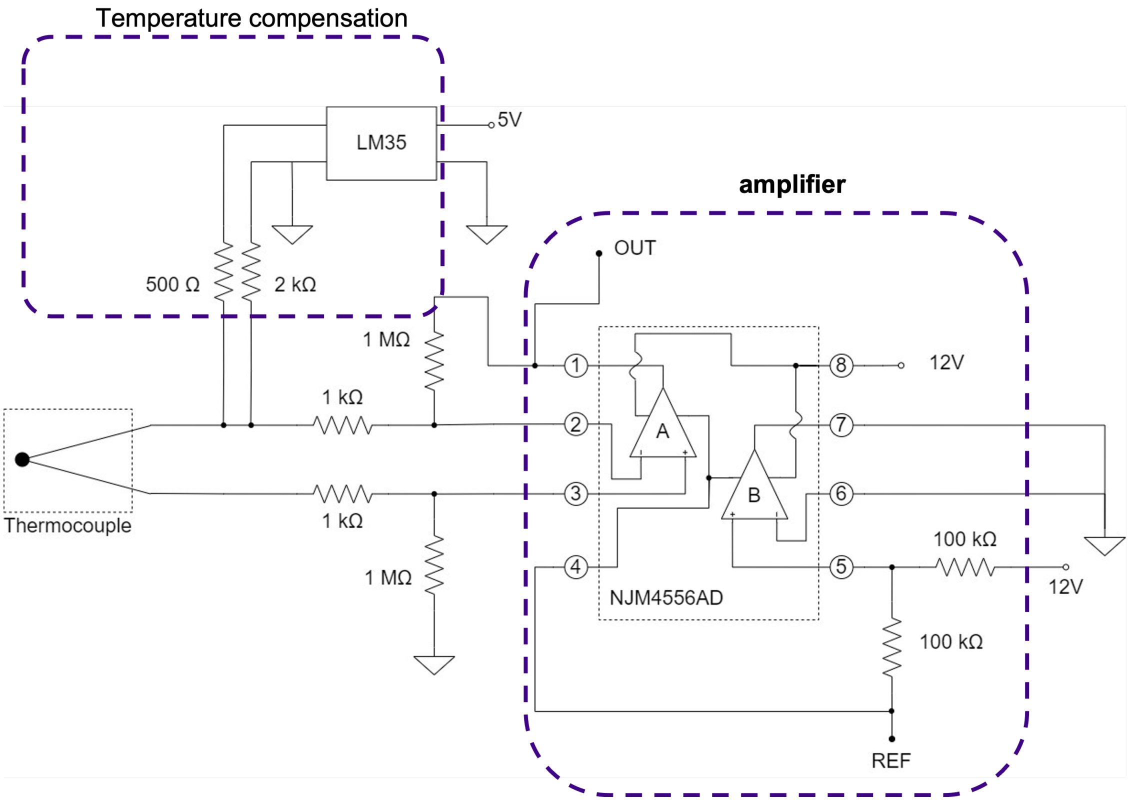

Figure 1: Schematic of the customized circuit for thermocouple voltage amplification and temperature compensation.

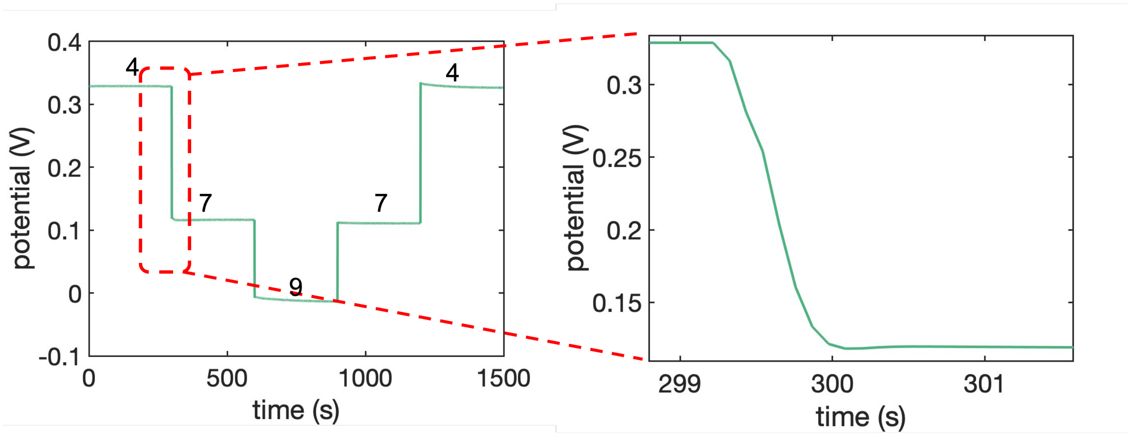

Figure 2: Our PANI-coated fibers for pH sensing in buffer solutions with pH values of 4, 7, and 9. The fibers exhibited a sensitivity of 68.6 mV/pH, along with a rapid response to changes in pH.

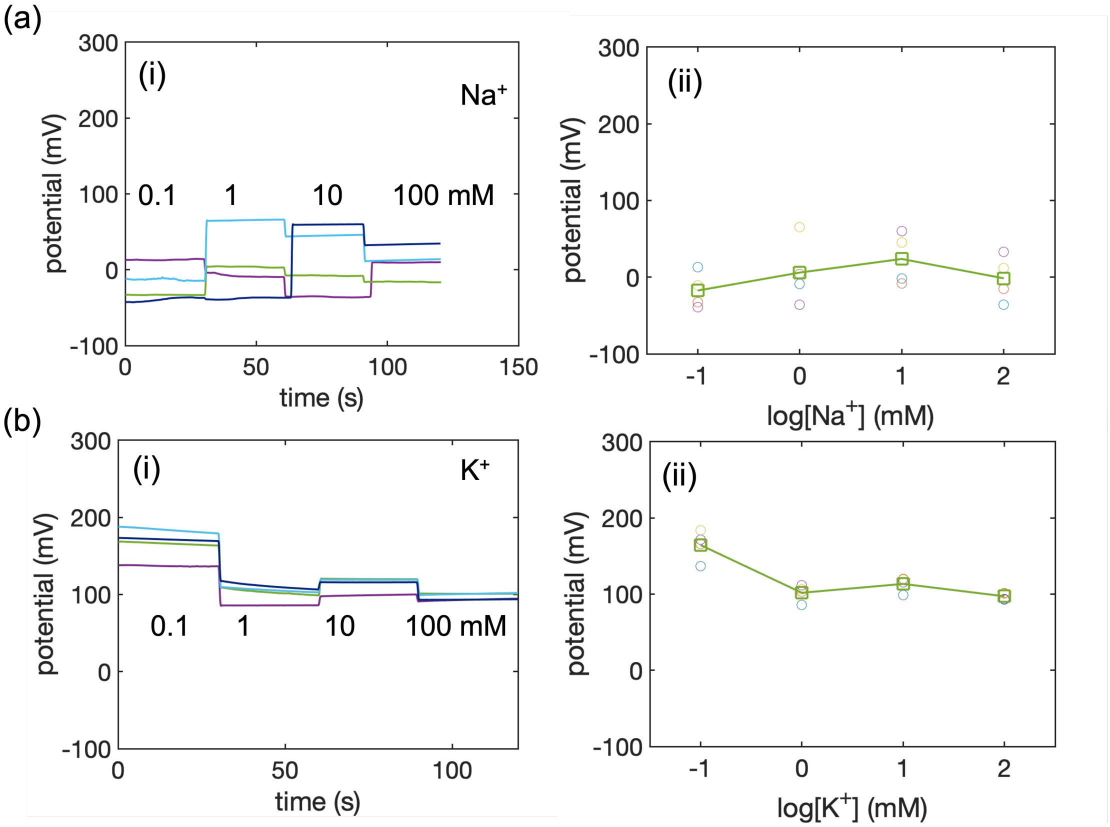

Figure 3: Characterization of ion selectivity of PANI-coated hybrid fibers. (a) Selectivity over Na^+. (b) Selectivity over K^+. The device in Figure 2 was subject to the selectivity measurement of Na^+ and K^+ for 4 times. In these experiments NaCl and KCl solutions were prepared over the concentrations of 0.1 mM, 1 mM, 10 mM, 100 mM.

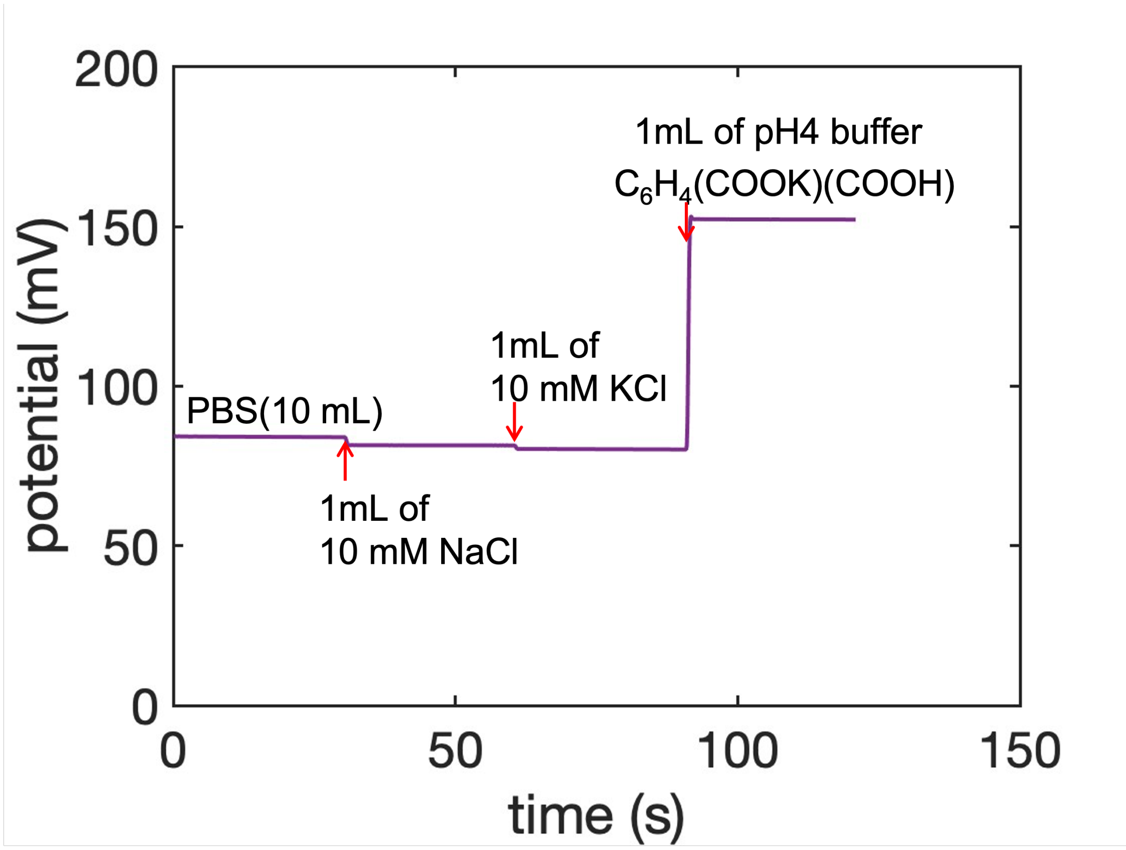

Figure 4: Characterization of ion selectivity of PANI-coated hybrid fibers in phosphate buffer solutions (PBS, 10 mL) with the addition of 1mL of 10 mM NaCl, 10 mM KCl and pH4 buffer solutions (same device). The addition of NaCl and KCl had minimal influence on the recorded open-circuit potential, indicating that PANI does not bind to Na^+ or K^+ ions. The pH of the PBS solution, initially recorded as 7.3 using a pH meter, changed to 7.3, 7.5, and 6.3 upon the addition of NaCl, KCl, and pH 4 buffer solutions, respectively. Only the addition of pH4 buffer solutions evoked the OCP increase of 71.1 mV which corresponds to the measured pH changes as well as indicated by sensitivity(68.6mV/pH)) of the sensors.

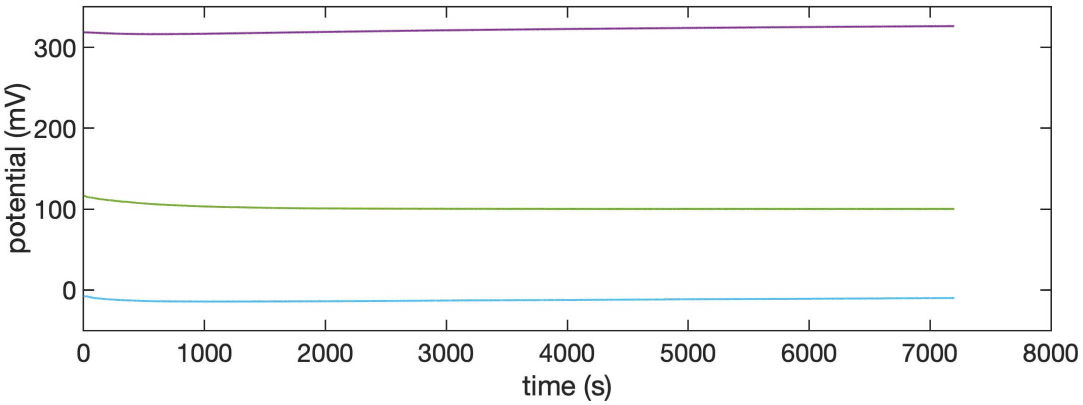

Figure 5: Voltage drift behaviors of our PANI-coated fibers in different pH buffer solutions of pH4 (purple), pH7 (green) and pH9 (blue) over 2 hours.

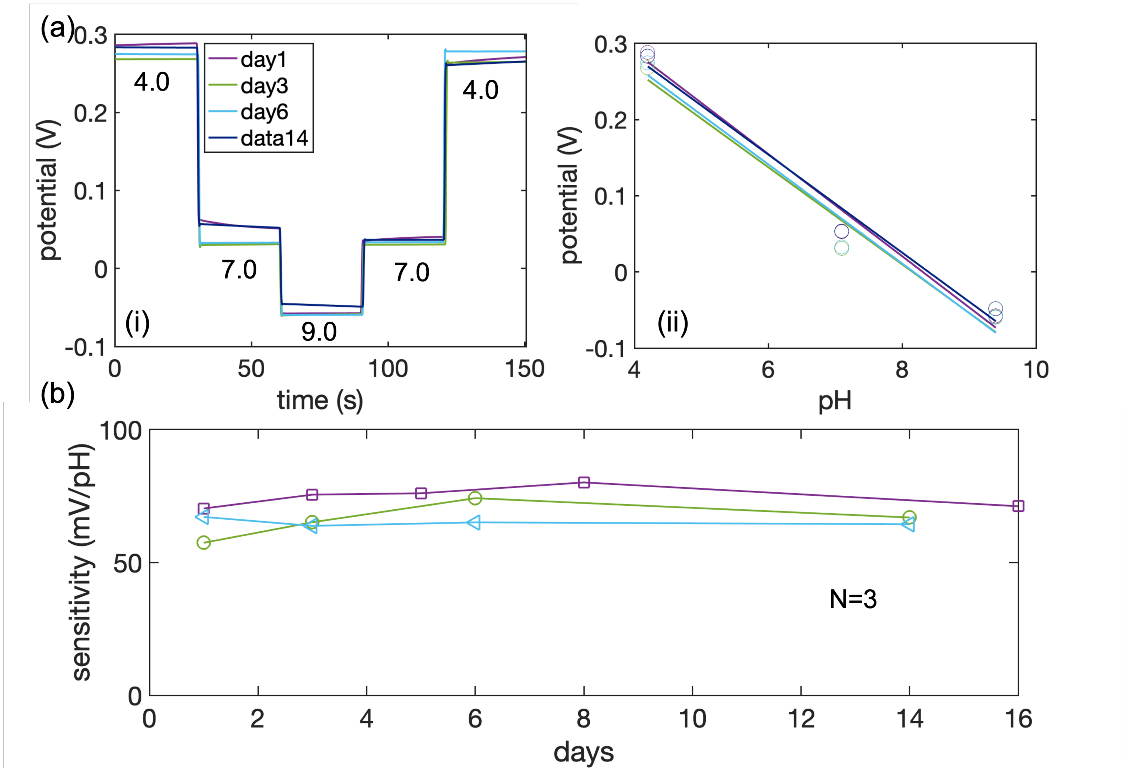

Figure 6: Long-term stability of PANI-coated hybrid fibers across different devices (N=3). (a) Example devices with pH sensing measurements and sensitivity over several days. (b) Sensitivity of multiple devices across different days.

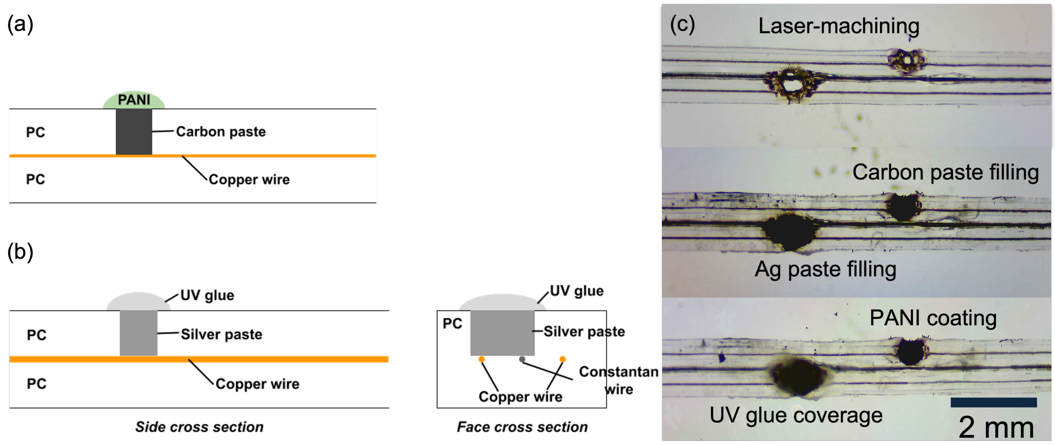

Figure 7: The schematics of the laser micromachining and surface functionalization of multi-wire fibers are shown in (a-b), along with microscopic images of the fiber after laser exposure and subsequent functionalization.
